# Ear Piercing Complications: Comparing Cartilage and Soft Tissue Piercings in a Large Survey Cohort

**DOI:** 10.1002/lary.70572

**Published:** 2026-04-17

**Authors:** John P. Ziegler, Nitin A. Pagedar, McKay Moline, Kathryn Marcus, Zachary G. Tanenbaum, Henry T. Hoffman, Scott R. Owen

**Affiliations:** ^1^ Department of Otolaryngology – Head and Neck Surgery University of Iowa Iowa City Iowa USA; ^2^ Colorado West Otolaryngologists Grand Junction Colorado USA; ^3^ Massachusetts Eye and Ear Department of Otolaryngology – Head and Neck Surgery Boston Massachusetts USA

**Keywords:** cartilage, complication, infection, piercing

## Abstract

**Objectives:**

To compare complication and removal rates between cartilage and lobule ear piercings in a large adult population.

**Methods:**

An anonymous electronic survey was distributed to all individuals with a University of Iowa email address. Respondents reported demographic characteristics, piercing site, age at piercing, technique, personnel, and specific complications. Multivariate logistic regression evaluated complication and removal rates between cartilage and lobule sites.

**Results:**

Data were analyzed on 9016 ear piercings from 3270 respondents, including 6275 (69.6%) lobule piercings and 2741 (30.4%) auricular cartilage piercings. Complications were reported in 40.2% of cartilage piercings versus 25.4% of lobule piercings (odds ratio [OR] 1.98, *p* < 0.0001). Infection occurred in 30.3% of cartilage piercings versus 23.8% of lobule piercings (OR 1.39, *p* < 0.0001). Additionally, cartilage site and presence of any complication each independently predicted piercing removal (OR 1.62, *p* < 0.0001; OR 12.82, *p* < 0.0001).

**Conclusion:**

Cartilage piercings carried significantly greater odds of complication and removal compared to lobule piercings. These findings underscore the importance of thorough counseling and informed consent, especially when piercing through auricular cartilage.

**Level of Evidence:**

3.

## Graphical abstract

Cartilage ear piercings were associated with significantly higher complication rates than lobule piercings and remained an independent predictor of complications after multivariate analysis. Cartilage site also independently predicted piercing removal, with higher removal rates observed compared to lobule piercings.

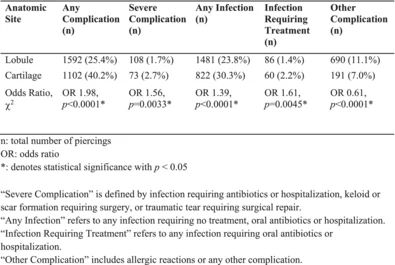

## 1 Introduction

Cosmetic ear piercing has long been a common cultural and aesthetic practice worldwide, with the lobule traditionally serving as the most common site. In recent decades, however, “high” or transcartilaginous ear piercings have grown in popularity, particularly among adolescents and young adults [1, 2, 3, 4, 5]. This trend has raised clinical concern due to the unique anatomical and physiological properties of auricular cartilage.

The avascular nature of the cartilage has been thought to limit antibiotic penetration and the ability to clear infection. This can predispose patients to serious complications including chondritis and perichondritis, which can result in abscess formation, necrosis, or deformity if not promptly and aggressively treated [5, 6, 7, 8, 9, 10, 11, 12]. *Pseudomonas aeruginosa* accounts for the majority of post‐piercing cartilaginous infections and can necessitate admission with parenteral antibiotics [4, 12, 13, 14]. Other common complications can include allergic reaction, embedding, traumatic tear, hematoma, hypertrophic scar, or keloid formation [3, 4, 6, 15].

A prior study performed at this institution [2] found that 35% of patients (30% of all sites) experienced a complication with ear piercing, though none were deemed serious or required hospitalization. Infection rates were found to be significantly increased in the cartilage piercings as compared to the lobule piercings (30% vs. 21%), though overall complication rates were similar.

Despite the growing popularity of transcartilaginous piercings, there is a paucity of similar comprehensive studies comparing complication rates between cartilage and lobule piercings. This study aims to fill this gap by systematically analyzing and quantifying complications of auricular piercings. To the authors' knowledge, this is the largest study to date directly comparing complication rates and removal outcomes by piercing site.

## 2 Materials and Methods

### 2.1 Study Design and Approval

This was a cross‐sectional survey‐based study conducted to evaluate complication rates associated with ear piercings at cartilage versus soft tissue (lobule) sites. The study protocol was initially approved by the University of Iowa Institutional Review Board (IRB #202004182) in 2020 with exempt status. Although the IRB approval expired before data analysis was completed, the project was resubmitted and granted a new exempt determination for the analysis of previously collected data.

### 2.2 Survey Distribution and Participants

An electronic survey was distributed via email to all University of Iowa email addresses. Participation was voluntary and anonymous. To incentivize participation, respondents were given the opportunity to enter a drawing to win one of four fifty‐dollar Amazon gift cards. Inclusion criteria required that participants be at least 18 years of age at the time of survey completion. Informed consent was obtained from participants.

### 2.3 Survey Content and Data Collection

The survey collected a wide range of information regarding each respondent's ear piercings. Participants provided demographic data including age, gender, and race or ethnicity. Respondents could report multiple piercings. For each piercing reported, respondents indicated the specific anatomical location category of the piercing, the age at which it was performed, the instrument or technique used, categorized as (1) piercing gun, (2) needle, or (3) other, and the type of personnel who performed the piercing, categorized as (1) physician or professional piercer, (2) hairdresser/beautician or store employee, or (3) self or friend.

**FIGURE 1:**
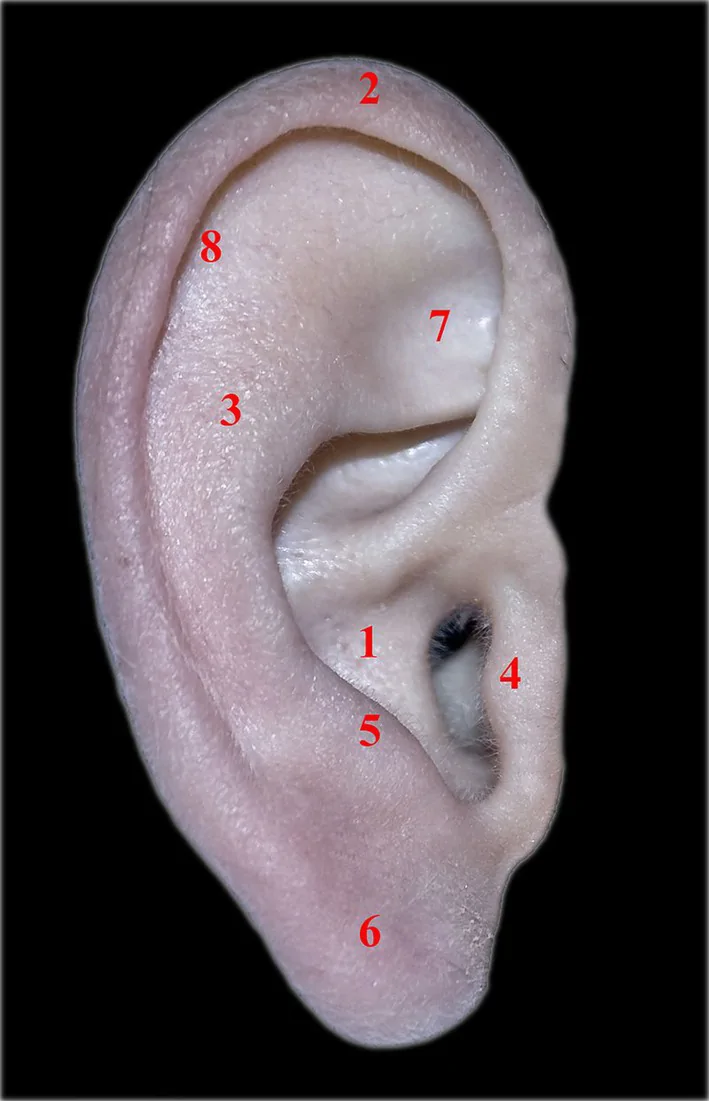
Labeled diagram of the external ear that was provided within the survey. Sites include: Concha (1), helix (2), anti‐helix (3), tragus (4), anti‐tragus (5), lobule (6), fossa triangularis (7), and scapha (8).[Color figure can be viewed in the online issue, which is available at www.laryngoscope.com]

To ensure anatomical clarity, a labeled diagram (Figure 1) of the external ear was provided within the survey, highlighting eight piercing sites: concha, helix, anti‐helix, tragus, anti‐tragus, lobule, fossa triangularis, and scapha. For the purposes of analysis, piercings at the concha, helix, anti‐helix, tragus, anti‐tragus, fossa triangularis, and scapha were categorized as cartilage piercings, while piercings at the lobule were categorized as lobule piercings. Participants were asked to report whether any complications occurred at each piercing site and to specify the nature of the complication using a checklist. Complication types included: infection not requiring medical attention, infection requiring antibiotics, infection requiring hospitalization, keloid or scar formation not requiring surgical intervention, keloid or scar formation requiring surgical intervention, traumatic tear of the ear not requiring surgery, traumatic tear of the ear requiring surgery, allergic reaction, or other. Respondents were also asked whether any piercings had been removed.

### 2.4 Statistical Analysis

Descriptive statistics were used to characterize demographic variables and the distribution of piercing sites and complications. Univariate analyses were conducted to assess the relationship between piercing location (cartilage) and any complication. The primary outcomes of interest in multivariate logistic regression were: (1) whether cartilage location independently predicted the occurrence of any complication, and (2) whether cartilage location independently predicted removal of the piercing. These models adjusted for potential confounders including age at the time of piercing, instrument or technique, personnel performing the piercing, gender, and race or ethnicity. Odds ratios (OR) with corresponding 95% confidence intervals were reported, and a *p*‐value of < 0.05 was considered statistically significant. All analyses were performed using SAS 9.4.

## 3 Results

A total of 3298 respondents completed the survey. After excluding 28 respondents under age 18 and their 64 piercings, the final analytic sample included 3270 respondents and 9016 ear piercings. Of these, 6275 were lobule piercings (69.6%) and 2741 were cartilage piercings (30.4%) as seen in Table 1.

**TABLE 1:** Cartilage versus lobule piercings.

Anatomical site	Number of piercings
Cartilage sites	
Concha	250 (2.8%)
Helix	452 (5.0%)
Antihelix	114 (1.3%)
Tragus	355 (3.9%)
Antitragus	97 (1.1%)
Fossa triangularis	179 (2.0%)
Scapha	1294 (14.4%)
Total	2741 (30.4%)
Soft tissue sites	
Lobule	6275 (69.6%)
Total piercings	9016

### 3.1 Description of the Cohort

Most respondents across both groups identified as White females. Cartilage piercings were more often performed later in life with mean age at piercing of 18.8 versus 9.7 years for lobule piercings. The primary instrument used for cartilage piercings was the needle compared to lobule piercings, where piercing gun use was most common. Professionals, including physicians and licensed piercers, performed the majority of cartilage piercings while the majority of piercings in the lobule group were performed by a hairdresser/beautician or store employee. Additional information can be found in Table 2.

**TABLE 2:** Respondent demographics, piercing technique, and removal by site.

	Lobule	Cartilage
Mean age at survey (years)	31.9 (range 18–80)	28.9 (range 18–69)
Gender (n)		
Male	241 (3.9%)	48 (1.8%)
Female	5981 (95.3%)	2656 (96.9%)
Other	53 (0.8%)	37 (1.3%)
Race or ethnicity (n)		
American Indian/Alaska Native	28 (0.4%)	9 (0.3%)
Asian	319 (5.1%)	99 (3.6%)
Black or African American	158 (2.5%)	59 (2.2%)
Native Hawaiian or Other Pacific Islander	12 (0.2%)	2 (0.1%)
White or Caucasian	5381 (85.8%)	2415 (88.1%)
Other	158 (2.5%)	68 (2.5%)
Hispanic or Latino	219 (3.5%)	89 (3.2%)
Instrument (n)		
Piercing gun	4694 (87.0%)	797 (30.1%)
Needle	650 (12.1%)	1817 (68.5%)
Other	51 (0.9%)	38 (1.4%)
Personnel (n)		
Physician/professional piercer	623 (11.9%)	1332 (52.8%)
Self/friend	239 (4.6%)	146 (5.8%)
Hairdresser/beautician or store employee	4365 (83.5%)	1046 (41.4%)
Mean Age at Piercing (years)	9.7 (SD 5.7, range 0–65)	18.8 (SD 5.8, range 0–59)
Removal of piercing (n)		
Yes	320 (5.1%)	265 (9.7%)
No	5924 (94.9%)	2469 (90.3%)
	OR 1.99, *p* < 0.0001	

*Note:* Instrument was unknown in 969 piercings. Personnel unknown in 1277 piercings. Removal unknown in 38 piercings. Odds ratio listed above is pertaining to removal of piercing compared between lobule and cartilage groups.

Abbreviations: *n*, total number of piercings; OR, odds ratio; SD, standard deviation.

### 3.2 Complications

Complications were significantly more common in cartilage piercings than lobule piercings with a complication of any kind reported in 1102 (40.2%) cartilage piercings compared to 1592 (25.4%) lobule piercings. Severe complication—defined as infection requiring antibiotics or hospitalization, keloid or scar formation requiring surgery, or traumatic tear requiring surgical repair—occurred significantly more often in cartilage piercings. Infection and infection requiring treatment both also occurred more often in cartilage piercings. Other complications—defined as allergic reaction or other—were seen more often in lobule piercings (Table 3).

**TABLE 3:** Piercing complications by site.

Anatomic site	Any complication (*n*)	Severe complication (*n*)	Any infection (*n*)	Infection requiring treatment (*n*)	Other complication (*n*)
Lobule	1592 (25.4%)	108 (1.7%)	1481 (23.8%)	86 (1.4%)	690 (11.1%)
Cartilage	1102 (40.2%)	73 (2.7%)	822 (30.3%)	60 (2.2%)	191 (7.0%)
Odds ratio, χ^2^	OR 1.98, *p* < 0.0001*	OR 1.56, *p* = 0.0033*	OR 1.39, *p* < 0.0001*	OR 1.61, *p* = 0.0045*	OR 0.61, *p* < 0.0001*

*Note:* “Severe Complication” is defined by infection requiring antibiotics or hospitalization, keloid or scar formation requiring surgery, or traumatic tear requiring surgical repair. “Any Infection” refers to any infection requiring no treatment, oral antibiotics or hospitalization. “Infection Requiring Treatment” refers to any infection requiring oral antibiotics or hospitalization. “Other Complication” includes allergic reactions or any other complication.

Abbreviations: *n*, total number of piercings; OR, odds ratio.

* Statistical significance with *p* < 0.05.

In univariate analysis, cartilage site was a strong predictor of complication with nearly double the odds of complication compared to lobule site (OR 1.99, 95% confidence interval (CI): 1.81–2.19, *p* < 0.0001). Piercing gun use was associated with a lower complication rate compared to other methods while piercings performed by physicians or professional piercers were associated with a higher odds of complication. Compared to White respondents, Black respondents had a significantly lower odds of complication, and males were associated with higher complication odds when compared to females (Table 4).

**TABLE 4:** Univariate analysis: prediction of complication by cartilage site.

	OR	95% CI, *p*	c‐stat
Cartilage site	1.99	1.81–2.19, < 0.0001*	0.576
Age (per year)	1.03	1.02–1.03, < 0.0001*	0.562
Instrument			0.538
Piercing gun	0.77	0.50–1.20, 0.01*	
Needle	1.09	0.70–1.70, 0.07	
Personnel			0.521
Physician/professional piercer	1.21	1.08–1.35, 0.0007*	
Self/friend	0.87	0.68–1.09, 0.044*	
Race or ethnicity			0.512
American Indian/Alaska Native	0.88	0.44–1.77, 0.99	
Asian	1.16	0.94–1.43, 0.05	
Black or African American	0.48	0.33–0.68, 0.001*	
Native Hawaiian or Other Pacific Islander	0.93	0.29–2.97, 0.91	
Other	0.95	0.71–1.27, 0.65	
Hispanic or Latino	0.95	0.74–1.21, 0.62	
Gender			
Male	1.11	0.86–1.42, 0.02*	0.507
Other	2.60	1.71–3.95, < 0.0001*	

*Note:* Univariate analysis determining if cartilage site predicts complication. All instruments compared against “Other” group. All personnel compared against “Hairdresser/Beautician or Store Employee” group. All races/ethnicities compared against “White or Caucasian” group. All genders compared against “Female” group.

Abbreviations: c‐stat, c‐statistic; CI, confidence interval; OR: univariate odds ratio.

* Statistical significance with *p* < 0.05.

After adjusting for variables including age at the time of piercing, instrument or technique, personnel performing the piercing, gender, and race or ethnicity, cartilage site remained the strongest independent predictor of complication (OR 2.20, 95% CI: 1.90–2.56, *p* < 0.0001). Additional significant independent predictors included gender, race or ethnicity, and personnel performing the piercing (Table 5).

**TABLE 5:** Multivariate analysis: prediction of any complication by variable.

	OR	95% CI	*p*
Cartilage site	2.20	1.90–2.56	< 0.0001*
Age (per year)	0.99	0.98–1.01	0.77
Instrument			0.98
Piercing gun	0.98	0.56–1.73	
Needle	0.97	0.56–1.68	
Personnel			0.04*
Physician/professional piercer	0.86	0.73–1.00	
Self/friend	0.74	0.56–0.97	
Race or ethnicity			0.03*
American Indian/Alaska Native	1.14	0.51–2.57	
Asian	1.41	1.11–1.81	
Black or African American	0.63	0.42–0.93	
Native Hawaiian or Other Pacific Islander	1.04	0.28–3.96	
Other	1.09	0.78–1.54	
Hispanic or Latino	0.96	0.71–1.30	
Gender			
Male	1.34	1.01–1.78	0.0004*
Other	2.22	1.41–3.50	

*Note:* All instruments compared against “Other” group. All personnel compared against “Hairdresser/Beautician or Store Employee” group. All races/ethnicities compared against “White or Caucasian” group. All genders compared against “Female” group.

Abbreviations: CI, confidence interval; OR: adjusted multivariate odds ratio.

* Statistical significance with *p* < 0.05.

### 3.3 Piercing Removal

Removal of piercings was more common among cartilage sites, with 9.7% of piercings removed compared to 5.1% of lobule piercings (Table 2). In univariate and multivariate analyses, cartilage site and presence of any complication predicted piercing removal (Tables 6 and 7). Any complication was the strongest independent predictor of removal (OR 12.82, 95% CI: 10.04–16.63, *p* < 0.0001).

**TABLE 6:** Univariate analysis: prediction of removal by cartilage site.

	OR	95% CI	*p*
Cartilage site	1.98	1.67–2.34	< 0.0001*
Age (per year)	1.007	0.996–1.02	0.20
Instrument			
Piercing gun	0.46	0.24–0.90	0.003*
Needle	0.63	0.32–1.25	0.71
Personnel			
Physician/professional piercer	1.20	0.98–1.47	0.46
Self/friend	1.17	0.78–1.76	0.74
Race or ethnicity			
American Indian/Alaska Native	0	< 0.001 to > 999	0.96
Asian	1.64	1.17–2.30	0.96
Black or African American	1.64	1.04–2.60	0.96
Native Hawaiian or Other Pacific Islander	0.88	0.54–1.45	0.97
Other	2.53	0.56–11.33	0.95
Hispanic or Latino	1.70	1.10–2.64	0.96
Gender			
Male	1.51	1.01–2.27	0.39
Other	1.43	0.69–2.98	0.69
Complication	11.84	9.58–14.63	< 0.0001*

*Note:* All instruments compared against “Other” group. All personnel compared against “Hairdresser/Beautician or Store Employee” group. All races/ethnicities compared against “White or Caucasian” group. All genders compared against “Female” group.

Abbreviations: CI, confidence interval; OR, univariate odds ratio.

* Statistical significance with *p* < 0.05.

**TABLE 7:** Multivariate analysis: prediction of removal by variable.

	OR	95% CI	*p*
Cartilage site	1.62	1.28–2.06	< 0.0001*
Instrument			0.099
Piercing gun	0.55	0.27–1.14	
Needle	0.48	0.23–0.99	
Complication	12.82	10.04–16.63	< 0.0001*

*Note:* All instruments compared against “Other” group.

Abbreviations: CI, confidence interval; OR, adjusted multivariate odds ratio.

* Statistical significance with *p* < 0.05.

## 4 Discussion

Complications following ear piercings are common, with reported rates varying widely depending on factors such as anatomical site, technique, and aftercare. Minor infections, allergic reactions, and keloid formation are among the most frequently reported issues and can occur at different rates based on the location. As transcartilaginous piercings have grown in popularity, they have been thought to be associated with increased complications as compared to lobule piercings, though few high‐quality studies exist [1, 2, 5, 6, 7, 8, 9, 10, 11, 13, 14, 16].

Our study supports this claim as we found increased rates of complication in cartilage piercings when compared to lobule piercings (40.2% and 25.4%, respectively). When controlling for other variables, cartilage piercings were found to have odds of any complication 2.2 times higher than lobule piercings. This finding strongly evidences cartilage piercing as an independent predictor of any complication.

Infection rates too were found to be significantly more common in the cartilage group (30.3%) as compared to the lobule group (23.8%). Simplot and Hoffman's 1998 study [2] found similar increased rates of infection in cartilage piercings (30% vs. 21%), though they did not report a higher overall complication rate as we found in our study. This might suggest that while infections occur more frequently in cartilage piercings, lobule piercings may still incur a high frequency of other minor issues like allergic reactions. Our study did find the lobule group to have significantly higher rates of allergic reactions or other complications (11.1%) compared to the cartilage group (7.0%), though cartilage still independently predicted any complication.

Serious complications following transcartilaginous piercing have also been commonly reported and can range from perichondritis or chondritis to systemic complications such as toxic shock syndrome or disseminated bacterial infections [4, 17, 18]. Our study found severe complications, defined as infection requiring antibiotics or hospitalization, keloid or scar formation requiring surgery, or traumatic tear requiring surgical repair, to occur in just 2.7% of cartilage piercings and 1.7% of lobule piercings. Only 2% of all piercings in our study required hospitalization or surgery, and 1.6% required antibiotics. These figures were mildly increased from Simplot and Hoffman's study, which noted no hospitalizations and just a 1% antibiotic rate. Other studies examining body piercings, not specific to the ear, have found similarly low rates of severe complications [1, 19].

We also observed a significantly higher rate of piercing removal in the cartilage group (9.7%) versus the lobule group (5.1%). In our multivariate model, cartilage site and the occurrence of any complication independently predicted removal. This adjusted finding possibly reflects the limited vascularity and poor healing potential of auricular cartilage compared to soft tissue, thus necessitating removal.

The role of the piercing instruments, and their relation to complications, remains debated. In our cohort, piercing guns were overwhelmingly used for lobule piercings (87%), whereas cartilage piercings were more often performed with needles (68.5%). This distribution is consistent with recommendations to avoid piercing guns for cartilage due to the theoretical risk of perichondral shearing and cartilage necrosis [8, 11, 20]. Conversely, one histological study found there to be limited objective evidence that piercing guns cause more structural damage than needles [21]. In our study, instrument type was not a significant predictor of complication or removal when assessed independently, again consistent with results from Simplot and Hoffman, though differing from those of Binkhamis et al. [11]. This may suggest that other factors may be more influential in determining outcomes.

Trends in piercing personnel have also evolved over time. In our study, professionals, including physicians and licensed piercers, performed the majority of cartilage piercings (52.8%), while the majority of lobule piercings were performed by a hairdresser/beautician or a store employee (83.5%). Personnel was found to independently predict the presence of complications in our multivariate analysis (*p* = 0.04). This difference may reflect greater public awareness of the risks associated with cartilage piercing as well as increased availability of professional services.

This study offers several strengths and a significant methodological advance over prior research. To our knowledge, it represents the largest dataset to date directly comparing complication and removal rates between cartilage and lobule piercings. However, several limitations must be acknowledged. First, the sample was reflective of the population at our large Midwestern university, with a predominance of White female respondents. While this represents improved diversity compared to other similar studies [2, 11], it still limits generalizability. Additionally, certain patient‐level factors, including smoking status and other medical comorbidities, were not examined. Second, our data are self‐reported, raising the possibility of recall bias and subjective reporting. We did not confirm infections, scarring, or other complications through clinical examination. Third, we were unable to control aftercare practices or aseptic techniques, which are known contributors to outcome variability. Despite these limitations, our findings reinforce the need for cautious consideration of cartilage piercing, and underscore the importance of informed consent, aftercare education, and stricter regulation of piercing practices.

## 5 Conclusion

Cartilage ear piercings are associated with significantly higher complication rates and removal rates than lobule piercings. These findings reinforce existing evidence that cartilage is more vulnerable to infection and poor healing, likely related to its limited vascularity. As cartilage piercings grow in popularity, especially among young adults, these data underscore the importance of pre‐procedural counseling and informed consent when considering piercing location.

## Funding

This work was supported by the Department of Otolaryngology—Head and Neck Surgery, University of Iowa (NIH/NCI P30CA086862 and UM1TR004403).

## Conflicts of Interest

The authors declare no conflicts of interest.

## Data Availability

The data that support the findings of this study are available on request from the corresponding author. The data are not publicly available due to privacy or ethical restrictions.
